# Heterocycles in Medicinal Chemistry

**DOI:** 10.3390/molecules24213839

**Published:** 2019-10-25

**Authors:** Josef Jampilek

**Affiliations:** 1 Department of Analytical Chemistry, Faculty of Natural Sciences, Comenius University, Ilkovicova 6, 842 15 Bratislava, Slovakia; 2 Regional Centre of Advanced Technologies and Materials, Faculty of Science, Palacky University, Slechtitelu 27, 783 71 Olomouc, Czech Republic

Heteroatoms constitute a very common fragment of a number of active pharmaceutical ingredients as well as excipients; from the point of view of significance, it is all the same if these are isosterically/bioisosterically replaced carbons/carbon substructures in aliphatic structures or real heterocycles. Many heterocyclic scaffolds can be considered as privilege structures. Most frequently, nitrogen heterocycles or various positional combinations of nitrogen atoms, sulphur, and oxygen in five- or six-membered rings can be found. According to statistics, more than 85% of all biologically-active chemical entities contain a heterocycle. This fact reflects the central role of heterocycles in modern drug design. The application of heterocycles provides a useful tool for modification of solubility, lipophilicity, polarity, and hydrogen bonding capacity of biologically active agents, which results in the optimization of the ADME/Tox properties of drugs or drug candidates. The increasing presence of various heterocycles in drugs is related to advances in synthetic methodologies, such as metal-catalyzed cross-coupling and hetero-coupling reactions, that allow rapid access to a wide variety of functionalized heterocycles. On the other hand, many heterocyclic lead compounds were isolated from natural resources, and their structures were subsequently simplified and modified by medicinal chemists. Thus, heterocycles have critical importance for medicinal chemists, because using them, it is possible to expand the available drug-like chemical space and drive more effective drug discovery programs. As medicinal chemistry is “a chemistry-based discipline, also involving aspects of biological, medical, and pharmaceutical sciences” and “concerned with the invention, discovery, design, identification, and preparation of biologically active compounds, the study of their metabolism, the interpretation of their mode of action at the molecular level and the construction of structure-activity relationships”, this Special Issue of *Molecules*, titled “Heterocycles in Medicinal Chemistry”, is devoted to the following research topics focused on heterocycles: (i) synthesis and analysis; (ii) natural compounds; (iii) carbohydrates; (iv) drug design; (v) in silico investigations; (vi) biological screening; (vii) chemical biology and biological chemistry; (vii) biomaterials; and in general, other topics related to heterocycles.

The complexity and wideness of this issue are documented by two review articles. The first is a comprehensive up-to-date review focused on cinnoline derivatives exhibiting a broad spectrum of pharmacological activities such as antibacterial, antifungal, antimalarial, anti-inflammatory, analgesic, anxiolytic, and antitumor [1], and the second one deals with the research and applications of nucleotide analogues, especially the second-generation bridged nucleic acids [2].

Highly specific articles both present fluorimetric studies on the crystal structure of the new polymorphic form of 3-aminoflavone [3] and the investigations of the dihydrogen bond in the aminoborane complex of a nicergoline intermediate [4]. Sulekova et al. published a study evaluating the release of 5-fluorouracil from unmodified and modified mesoporous silica matrices [5]. A complex synthesis–analytical–galenical study described the synthesis of several piperazine-2,5-dione derivatives and their transdermal enhancement activity using theophylline as a model drug, and the prepared compounds can be potentially used as excipients in semi-solid drug formulations or transdermal therapeutic systems [6].

A unique screening of new potential drugs is represented by Awad et al. who focused on the design, synthesis, molecular modeling, and biological evaluation of novel 6-n-propyl-2-thiouracil analogues showing a promising anti-thyroid activity profile in reducing levels of thyroid hormones [7]. New histone deacetylases inhibitors derived from thiazolyl–coumarins have been designed by Pardo-Jiménez et al. Disulfide and hydroxamic acid derivatives were the most potent ones. The compounds exhibited antiproliferative effects and triggered a strong decrease in the expression levels of both α-smooth muscle actin and procollagen I; thus, these compounds decreased profibrotic effects on cardiac fibroblasts [8].

Psychiatric and neurodegenerative diseases are on the rise. Most neurodegenerative diseases cannot be cured; only their symptoms can be mitigated, or their progression hampered. Osmaniye et al. have reported the synthesis of novel thiazolylhydrazone derivatives as potential agents affecting Alzheimer’s disease by significant selective inhibition of acetylcholinesterase activity [9].

A publication on pharmacological topics presents both the search for mechanisms of actions of naphthoquinone-based chemotherapeutics active against acute myeloid leukemia [10] and studies evaluating cryptopleurine (thienoquinolizidine derivatives and (epi-)benzo analogues) phenanthroquinolizidine alkaloids inducing cell death of leukemia cells [11].

Several piperazine derivatives of ursolic acid analogues synthesized by Wang et al. as anticancer agents showed the IC_50_ values approx. 2 µM on the Hela and MKN45 cell lines. In addition, the compounds induced apoptosis, decreased the apoptosis regulator (BCL2/BAX) ratio, disrupted mitochondrial potential, and suppressed the growth of Hela xenografts in nude mice [12]. A series of four possible A-ring amino derivatives of luotonin A, a topoisomerase I inhibitor, obtained by intramolecular cycloadditions and catalytic transfer hydrogenation, was evaluated for antiproliferative activity towards human tumor cell lines. It was found that the compound significantly arrested G2/M cell cycle, which means that inhibition of topoisomerase I is not the only biological target [13]. Synthesis of 2,6-diamino-substituted purine derivatives as analogues of reversine and their screening against breast and colorectal cancer cells and affecting the cell cycle was described by Bosco et al. The compounds caused a cycle arrest in the G2/M phase. It should be noted that the compounds were effective only in cells, where p53 was deleted or down-regulated [14].

In addition to cancers, the increasing resistance of microbial pathogens to existing drugs remains a global problem. Therefore, the design of new antimicrobial drugs is a major challenge. Based on the naturally occurring compounds khellinone and visnagenone, new furothiazolo pyrimido quinazolinones were synthesized and screened on their antimicrobial activities. Several compounds demonstrated promising antibacterial and antifungal activities [15]. Old drugs become a great inspiration for newly synthesized molecules. Based on the structures of old agents, Malik et al. published a series of dibasic derivatives of phenylcarbamic acid significantly effective against various mycobacterial strains [16]. A series of 1-amino-2-aryliden- amine-1,2-(dicyano)ethenes as antifungal agents effective against yeasts were reported by Bettencourt et al. [17]. Several synthesized long chain alkyl amides of 2-amino-4-quinolones demonstrated a high inhibition of biofilm formation of *Pseudomonas aeruginosa* [18].

Polypharmacology and multi-target drugs are a very popular approach in medicinal chemistry. True to this approach, Lasak et al. prepared a new series of phenanthridines derived from benzo[*c*]phenanthridine alkaloids. Several compounds expressed high antibacterial activity against *Bacillus subtilis*, *Micrococcus luteus*, and/or *Mycobacterium vaccae*, and some derivatives also showed cytotoxicity against K-562 and MCF-7 cancer cell lines. The anti-tumor effective compounds caused an arrest of cell-cycle, apoptosis-specific fragmentation of PARP-1 and increased the levels of p53 protein [19].
